# Current Advances in Zika Vaccine Development

**DOI:** 10.3390/vaccines10111816

**Published:** 2022-10-28

**Authors:** Yuchen Wang, Lin Ling, Zilei Zhang, Alejandro Marin-Lopez

**Affiliations:** 1 Department of Inspection and Quarantine Technology Communication, Shanghai Customs College, Shanghai 201204, China; 2 Section of Infectious Diseases, Department of Internal Medicine, Yale University School of Medicine, New Haven, CT 06420, USA

**Keywords:** Zika virus, Zika vaccines, DNA vaccines, mRNA vaccines, live attenuated, whole inactivated, virus-like particles

## Abstract

Zika virus (ZIKV), an emerging arthropod-borne flavivirus, was first isolated in Uganda in 1947 from monkeys and first detected in humans in Nigeria in 1952; it has been associated with a dramatic burden worldwide. Since then, interventions to reduce the burden of ZIKV infection have been mainly restricted to mosquito control, which in the end proved to be insufficient by itself. Hence, the situation prompted scientists to increase research on antivirals and vaccines against the virus. These efforts are still ongoing as the pathogenesis and immune evasion mechanisms of ZIKV have not yet been fully elucidated. Understanding the viral disease mechanism will provide a better landscape to develop prophylactic and therapeutic strategies against ZIKV. Currently, no specific vaccines or drugs have been approved for ZIKV. However, some are undergoing clinical trials. Notably, different platforms have been evaluated for the design of vaccines, including DNA, mRNA, viral vectors, virus-like particles (VLPs), inactivated virus, live attenuated virus, peptide and protein-based vaccines, passive immunizations by using monoclonal antibodies (MAbs), and vaccines that target vector-derived antigens. These vaccines have been shown to induce specific humoral and cellular immune responses and reduce viremia and viral RNA titers, both in vitro and in vivo. This review provides a comprehensive summary of current advancements in the development of vaccines against Zika virus.

## 1. Introduction

Zika virus (ZIKV) is an arthropod-borne virus with the genus Flavivirus of the *Flaviviridae* family of enveloped RNA viruses. ZIKV has an ∼11 kb positive sense RNA genome. Translation of viral RNA in the cytoplasm generates a polyprotein that is cleaved into three structural proteins (capsid (C), pre-membrane/membrane (prM/M), and envelope (E)) and seven non-structural proteins (NS1, NS2A, NS2B, NS3, NS4A, NS4B, and NS5) [1]. ZIKV buds into the lumen of the endoplasmic reticulum as an immature virion composed of 60 icosahedrically arranged prM-E heterotrimers [2]. The ZIKV E protein is composed of three ectodomains (DI, DII, and DIII) and is the primary target of neutralizing antibodies (nAbs) [3]. ZIKV was first isolated from a non-human primate in 1947 in Uganda [4], and ZIKV infections in humans were sporadic for half a century before emerging in the Pacific and the Americas [5]. ZIKV is usually transmitted by the bite of infected *Aedes aegypti* mosquitoes, but sexual and vertical transmission has also been reported [6,7]. About two billion people worldwide live in tropical and sub-tropical regions with suitable environmental conditions for ZIKV [8], and increased globalization continues to raise the risk for disease spread.

The clinical presentation of Zika fever is nonspecific and can be misdiagnosed as other infectious diseases, especially those due to arboviruses such as dengue and chikungunya [9]. The outbreak of ZIKV threatens public health worldwide, especially as it can cause devastating congenital syndrome in the fetuses of pregnant women [10], including microcephaly, craniofacial disproportion, spasticity, ocular abnormalities, and miscarriage. In adults, Zika infection has been linked to the autoimmune disorder Guillain–Barré syndrome [11]. Prevention of congenital Zika syndrome (CZS) is the primary goal for immunization, and the vaccine must provide protection against intrauterine transmission for use during pregnancy and in women of childbearing age [12]. Ideally, a vaccine should also prevent sexual transmission of the virus through mucosal protection [13]. No vaccine is yet available for the prevention or treatment of Zika virus infection [14]. Despite the current waning in newly reported Zika infections, an efficacious vaccine is urgently needed to help limit the emergence of another detrimental epidemic. For these reasons, development of a Zika vaccine remains an active area of research [10].

The World Health Organization (WHO) and the United Nations International Children’s Emergency Fund (UNICEF) has issued a ZIKV vaccine target product profile (TPP), which outlines the desired characteristics and requirements for vaccines to protect against ZIKV [15]. This document makes special emphasis on generating efficient vaccines that confer protection against congenital Zika syndrome, especially during public health emergencies or outbreaks [16]. Significant scientific work has resulted in multiple candidate vaccines that are now undergoing further clinical development, with several candidates now in phase II clinical trials. In this review, we survey current vaccine efforts, preclinical and clinical results, and ethical and other concerns that directly bear on vaccine development. A wide variety of formulations are being studied, and more than 50 ZIKV vaccine candidates are now in various stages of research and development [17]. Since 2016, a number of candidates using multiple vaccine platforms have been developed and have shown promising results in preclinical testing [18]. Candidate ZIKV vaccines that are currently in phase I/II clinical trials include live virus vaccines, inactivated, subunit-based, viral vector vaccines, DNA–RNA vaccines, and even vaccines based on mosquito salivary antigens [19]. One or more pivotal Phase III trials is normally used to demonstrate safety and efficacy [20]. The next step would be Phase III field efficacy trials, which would not be possible since its peak in early 2016 [21], as the incidence of Zika virus (ZIKV) cases has declined [22]. The rapid progress in vaccine development demonstrates the capacity of governments, public health organizations and the scientific community to respond to the threat of a pandemic [23]. Despite low levels of transmission during recent years, ZIKV has become endemic in the Americas and the potential for large Zika outbreaks remains real [24]. The development of a safe and effective ZIKV vaccine is therefore an urgent global health priority [5], It is important for vaccine researchers to continue developing and improving Zika vaccines, so that a potential vaccine is ready for deployment and clinical efficacy trials when the next ZIKV outbreak occurs [18].

An effective and protective vaccine relies on several requirements, which include the following: (i) induction of specific immune response against immunodominant antigens [25]; (ii) selection of adjuvant–antigen formulation [26]; and (iii) assessment of safety, effectiveness, and long-term protection [27]. Based on prior flavivirus vaccine development programs, knowledge of flavivirus particle structure, definition of E dimers as a key antigenic target [28], and deep understanding of neutralizing mechanisms, multiple vaccine strategies have advanced to the stage of clinical evaluation with unprecedented speed. In this review, we will address the state of the art of the different vaccine platforms used against Zika infection.

## 2. ZIKV DNA Vaccines

The DNA vaccine platform has been used for over twenty-five years to create candidate vaccines against numerous pathogens, such as West Nile virus (WNV), infectious hematopoietic necrosis virus (IHNV), Ebola virus (EBOV), Rift Valley fever virus (RVFV), dengue virus (DENV), and chikungunya virus (CHIKV), and severe acute respiratory syndrome coronavirus 2 (SARS-CoV-2) [29,30,31,32,33,34,35]. DNA vaccines are generated by cloning an antigen(s) from a pathogen into a DNA plasmid, which will be further injected [36]. The initial safety and immunogenicity of a DNA vaccine encoding consensus ZIKV premembrane and envelope antigens delivered by electroporation has been shown to generate cellular and humoral immune responses, including the production of nAbs, in mice and non-human primates [37].This vaccine candidate afforded complete protection against a lethal challenge of ZIKV and induces a high level of specific antibody titers in mice [38]. In addition, the DNA vaccine platform carries virtually no risk of causing disease, since they deliver only a small portion of the full pathogen genome [39]. DNA vaccines can be quickly designed and manufactured at a low cost and are relatively stable and safe for adults and fetuses, avoiding virulence recovery induced by reproducible vaccines [40].

In addition, a DNA-based vaccine platform using two DNA vaccine candidates, VRC5288 and VRC5283, was developed by the Vaccine Research Center (VRC) of the National Institute of Allergy and Infectious Diseases (NIAID), National Institutes of Health (NIH), Bethesda, MD, USA. These candidates encode the protein prM (prM) and envelope protein E (E), respectively, and have completed clinical trials (Phases I and II; these trials are registered with ClinicalTrials.gov, numbers NCT02840487, NCT02996461 and NCT03110770) [41]. The results obtained from the immunization of non-human primates provide sufficient evidence to show that VRC5283 was well tolerated and to support clinical studies of VRC5283 in regions with endemic Zika virus to assess efficacy in humans [41]. In addition, C57BL/6 and IFNAR1 (deficient for IFN receptor I) mice inoculated (intramuscularly, by jet injection or by electrophoration (EP)) with a DNA vaccine candidate expressing tandem repeated ZIKV envelope domain III (ED III × 3), showed a significant induction of humoral and cellular immunity, by measuring nAbs and IFN-γ secretion from splenocytes, respectively. Among these, the EP delivery system enhanced the immunogenicity and protective efficacy of these DNA vaccines [42], as more robust ZIKV E specific-total IgG and nAbs, as well as T-cell responses were observed. Another DNA-based candidate, GLS-5700, contains a synthetic ZIKV prM-E consensus sequence antigen created by aligning the prM and E sequences of multiple ZIKV isolates collected between 1952 and 2015. A129 mice (also deficient for IFN receptor I) single immunized with GLS-5700 were fully protected from morbidity and mortality following ZIKV challenge. This vaccine also lowered viral loads in blood, semen, and other tissues and conferred protection of male mice from ZIKV-induced testicular damage, which suggests that it may also prevent sexual transmission [43]. Other viral antigens have been tested using DNA vaccine technologies, such as ZIKV non-structural protein NS1. A novel DNA vaccine encoding a secreted ZIKV NS1 confers rapid protection from systemic ZIKV infection in immunocompetent mice. The results show that functional NS1-specific T-cell responses are critical for protection against ZIKV infection [44]. The rapid development timeline of these vaccines further highlights the potential of the DNA vaccine platforms for meeting the challenge of developing therapies to stop the spread of potential Zika outbreaks and other emerging infectious diseases. 

## 3. ZIKV mRNA Vaccines

mRNA vaccine technology has arisen as a simplified, flexible, and fast vaccine production platform [45]. The main advantage of mRNA-based vaccines is the ability to be rapidly modified in order to eliminate undesired side effects or to enhance immunogenicity, e.g., to respond to mutations and antigenic changes in the organism [46]. mRNA has emerged as a versatile and highly effective platform to deliver vaccine antigens and therapeutic proteins [47]. mRNA vaccines can be synthesized with virtually any sequence [48], and modified mRNA molecules encoding specific mutations may abolish immunodominant cross-reactive epitopes in the E protein sequence (especially in the domain II fusion loop, E-DII-FL) [49]. A versatile ZIKV vaccine platform in which lipid nanoparticles encapsulate modified mRNA (mRNA-LNP) encoding ZIKV structural genes has been generated [50]. Following a prime-boost immunization of modified mRNA encoding ZIKV prM-E genes that produced virus-like particles resulted in high levels of nAbs that protected immunocompetent (C57BL/6) and immunocompromised (AG129, deficient in interferon alpha/beta/gamma receptors) mice [49]. These mRNA induced protective antibody responses against ZIKV in mice and minimized the generation of cross-reactive antibodies that enhanced DENV infection in cell culture and pathogenicity in mice [51]. Additionally, a single low dose intradermal immunization with nucleoside-modified mRNA-LNP encoding the pre-membrane and envelope (prM-E) glycoproteins of ZIKV from the 2013 outbreak elicits rapid and durable protective immunity in mice and non-human primates, therefore representing a new and promising vaccine candidate for the global fight against ZIKV [52]. Another mRNA vaccine candidate, that encodes the prM-E glycoproteins of ZIKV Brazil strain SPH2015, induced protective antibody responses in AG129 mice. These results showed that a single administration of ZIKV prM-E mRNA-LNP protected against a lethal dose of ZIKV, while a prime-boost strategy induced strong protective immunity [53]. Another platform, based on a self-amplifying messenger RNA (SAM) platform technology delivered by cationic nanoemulsion (CNE), has been shown to be clinically safe, and to be immunogenic in a variety of animal models and well tolerated in humans. This platform allows bedside mixing to develop the ZIKV vaccine candidates, which is also particularly useful for rapid responses to pandemic outbreaks [54]. Using this platform as a ZIKV vaccine candidate, potent neutralizing antibody responses to ZIKV in mice and non-human primates [55] were observed, providing a preclinical proof of concept that can rapidly elicit protective immunity against ZIKV [56].

Due to the existing critical situation, the mRNA vaccine approach has recently been developed to address COVID-19 disease and has been granted Emergency Use Authorization in the USA and Europe (Pfizer/BioNTech and Moderna, December 2020) [57]. In addition to COVID-19, Moderna has advanced its Zika vaccine candidate (mRNA-1893, phase I trials are registered with ClinicalTrials.gov, numbers NCT04064905), the preclinical data have shown that vaccination with mRNA-1893 protected against Zika virus transmission during pregnancy in mice [58], and currently it is being studied in a phase II clinical trials in United States and Puerto Rico (the phase II trials are registered with ClinicalTrials.gov, numbers NCT04917861), being developed in collaboration with the Biomedical Advanced Research and Development Authority (BARDA). With the success of mRNA vaccine technology in facing the coronavirus (COVID-19) pandemic, the development of mRNA vaccine against ZIKV and other infectious diseases will be reinforced, as a promising alternative to conventional vaccine approaches [59].

## 4. ZIKV Viral Vector Vaccines

A wide variety of viral vector vaccines have been generated and optimized against Zika infections [60]. Adenovirus (Ad) vectors are amenable to rapid, inexpensive scale-up since this is easy to engineer [61] and, most importantly, do not require specialized cold-chain storage, which make them an ideal platform for equitable global distribution or stockpiling [62]. At the same time, adenoviruses are relatively safe and highly immunogenic vaccine vectors capable of inducing long-lasting humoral and cellular immune responses [63]. Adenovirus type 5 (Ad5) has been the most extensively used vector due to the robust immune response elicited [64]. Adenovirus (Ad) vectored Zika virus vaccines work by inserting a ZIKV prM-E gene expression cassette into human Ad types 4 (Ad4-prM-E) and 5 (Ad5-prM-E). Ad5-prM-E elicited both strong antibody and T-cell responses, whereas another adenoviral vector, Ad4-prM-E, did not induce any detectable anti-ZIKV antibodies, although it still induced a strong T-cell response when it presented the same antigens, in female Ifnar1^−/−^ mice [65]. After a lethal ZIKV challenge in this mouse model, Ad5-prM-E provided superior protection to Ad4-prM-E vaccination. This study shows that both human Ad5 and Ad4 expressing the ZIKV full-length prM-E genes are candidates for ZIKV vaccine [65]. Another study also showed that Ad5-prM-E was able to induce both cell-mediated and humoral immune responses to ZIKV in immunocompetent BALB/c mice [66]. This adenovirus-based vaccine expressing ZIKV proteins was immunogenic and protective in mice, and it encoded ZIKV proteins in a conformation recognized by the human antibody repertoire [67]. Ad26.ZIKV.M-Env, an adenoviral vector type 26 (Ad26) that encodes the ZIKV M-E antigens, also induced strong and durable cellular and humoral immune responses in preclinical models. These humoral responses were characterized by envelope-binding and ZIKV neutralizing antibody responses, while cellular responses were characterized by ZIKV reactive CD4^+^ and CD8^+^ T cells [68]. Additionally, a phase 1 clinical testing of an Ad26.ZIKV.M-Env vaccine candidate (Ad26.ZIKV.001) is prompted. The high safety and immunogenicity profile makes Ad26.ZIKV.001 a promising ZIKV vaccine candidate for further development if the need reemerges [69], supporting the use of adeno vectors as a platform for ZIKV vaccine development [70]. Another adenovirus-based vector, the chimpanzee adenoviral vector (ChAdOx1), was used as a vaccine candidate against Zika infection following a single dose immunization. ChAdOx1-EDIII was shown to partially protect BALB/c mice upon an intravenous ZIKV challenge of 100 PFU, in which reduced and delayed levels of viremia were observed, compared with the control animals. A similar experiment was performed using the Ifnar1^−/−^ model, although no significant differences were observed regarding survival and viral titers in blood and tissues [71]. However, potential ways of optimization could be implemented in order to increase the efficacy of this candidate, such as increasing the dose of the vector or using multiple dose immunization (prime-boost).

Poxvirus-based vectors have been extensively used as vaccine candidates [72]. Recombinant poxvirus vector systems have a number of attractive features for vaccine development including a large payload capacity (at least 25,000 base pairs), potential for cold chain-independent distribution, lack of vaccine DNA integration, and induction of both cellular and humoral immunity [73]. An extensive history of poxvirus vector development has led to the first licensed recombinant poxvirus-based vaccine for human use (Modified Vaccinia Ankara (MVA) is an attenuated poxvirus that is frequently used as an Ebola viral vector, MVA-BN-Filo), with several others in late-stage clinical trials [74]. A large series of recombinant MVA (rMVA) vaccines have been evaluated in non-human primate (NHP) studies and in human clinical trials (these trials are registered with ClinicalTrials.gov, numbers NCT04028349, NCT00252148 and NCT01913353) [73,75,76,77]. For ZIKV, the immunogenicity and efficacy of a novel ZIKV vaccine is based on the highly attenuated poxvirus vector MVA expressing the ZIKV prM and E structural genes (termed MVA-ZIKV). Immunization of mice with MVA-ZIKV elicited antibodies that were able to neutralize ZIKV and induced potent and polyfunctional ZIKV-specific CD8^+^ T-cell responses that were mainly of an effector memory phenotype. Moreover, a single dose of MVA-ZIKV significantly reduced viremia in susceptible immunocompromised mice challenged with live ZIKV [78]. A single intramuscular immunization of immunocompetent mice with a ZIKV vaccine based on the ZIKV NS1 protein from a clinically proven safe, MVA vector (MVA-ZIKV-NS1 vaccine candidate) provided robust humoral and cellular responses, and afforded 100% protection against a lethal intracerebral dose of ZIKV (strain MR766) [79]. The NS1 protein itself has been shown to be a viable vaccine target, and it has been demonstrated that an attenuated recombinant vesicular stomatitis vector (rVSV)-based vaccine expressing ZIKV prM-E-NS1 as a polyprotein is a promising vaccine candidate for protection against ZIKV infection, highlighting an important role for NS1 in ZIKV-specific cellular immune responses [80]. This study demonstrates that the Zika virus NS1 protein is highly immunogenic and can elicit protective antibodies, underscoring its potential for an effective Zika virus vaccine, suggesting that the antibody response against the Zika virus NS1 protein is long-lasting and functionally active [81].

Another poxviral platform based on the multiplication-defective Copenhagen strain of vaccinia virus (VACV) (Sementis Copenhagen Vector (SCV)) was developed as a vaccine technology and assessed in non-human primates [72]. In this work, Indian rhesus macaques (*Macaca mulatta*) were vaccinated with a multi-pathogen recombinant SCV vaccine encoding the structural polyproteins of both ZIKV and CHIKV (SCV-ZIKA/CHIK). Vaccinated animals showed a significant reduction in viremia compared with animals that had received a control SCV inoculation, showing an effective induction of immunity in this animal model and illustrating the utility of SCV as a multi-disease vaccine platform capable of delivering multiple large immunogens.

## 5. ZIKV Virus-Like Particles (VLPs) Vaccines

To date, multiple virus-like particle (VLP)-based vaccine candidates have been created against ZIKV and have demonstrated the potential of the VLP vaccine platform as a cost-effective, highly protective and safe ZIKV vaccination strategy [82,83]. They have been shown to confer strong protective cellular immunity against ZIKV in mice [84].

The delivery of VLPs, based on the use of structural proteins [85], can be achieved by different approaches including DNA (e.g., NIAID/VRC) and mRNA (e.g., Moderna). The NIAID/VRC DNA vaccine encodes sequences from the French Polynesian isolate strain H/PF/2013 [40], and the Moderna mRNA vaccine encodes sequences from the Asian ZIKV strain Micronesia 2007 [49]. This VLP was generated in vitro for use as a vaccine following introduction of plasmid DNA encoding Zika structural protein (prM-E) genes into mammalian cells. There are several studies that show an efficient generation and purification of ZIKV VLPs [86]. One of these studies showed that VLPs produced in HEK293 mammalian cells using the prM and E structural proteins, when aluminum-adjuvanted, were able to induce nAbs in both mice and non-human primates and protected against ZIKV challenge [87]. Other forms of ZIKV VLPs have been also reported, featuring the co-expression of the prM-E, prM-E-NS1, C-prM-E, and NS2B/NS3 viral genes in human cells [88]. These studies show a strategy to assemble Zika VLPs by co-expressing the structural (C-prM-E) and non-structural (NS2B/NS3) proteins, showing that VLP immunizations elicited higher titers of nAbs, compared with the titers after inactivated Zika virus vaccination [82].

A complication for flavivirus vaccine development is the potential of immunogens to enhance infection via antibody-dependent enhancement (ADE), as ZIKV infection can generate poorly neutralizing and cross-reactive antibodies targeting the highly conserved fusion loop in EDII (EDII-FL), that may enhance DENV infection, based on some in vitro dose-dependent studies [89,90]. Approaches to avoid ADE are an important focus in the development of ZIKV vaccines. To minimize the effect of the cross-reactive ADE-facilitating antibodies between ZIKV and DENV, several novel mutations have been reported, either in or near the fusion loop (FL) of DII or DIII, to dampen the production of cross-reactive antibodies [91]. Immunization with a Zika VLP vaccine candidate containing the CD loop sub-structural domain from ZIKV E protein Domain III demonstrated immunoprotection in a murine model of ZIKV infection, stimulating protective antibodies associated with antibody-dependent cellular cytotoxicity (ADCC) and complement-dependent cytotoxicity (CDC) activities, representing a safer vaccine for preventing ADE [92].

## 6. ZIKV Purified Inactivated Vaccine

Inactivated vaccines provide enhanced safety at the cost of reduced immunogenicity, although they often require multiple doses and periodic boosters [93]. Despite the greatest need for the vaccine in pregnant women, this population is typically reserved for the final stages of clinical evaluation, given concerns for potential unforeseen adverse effects on the mother and developing fetus. However, inactivated virus vaccine platforms have a long track record of safety in both pregnant women and fetuses [12,94]. However, inactivated virus vaccine platforms have a long track record of safety in both pregnant women and fetuses [95]. A purified formalin-inactivated ZIKV vaccine (ZPIV) derived from a 2015 Puerto Rican ZIKV strain (PRVABC59; ZIKV-PR) similar to the highly circulating 2015 Brazilian strain (SPH2015; ZIKV-BR) [96] demonstrated protective efficacy in mice and non-human primates in preventing viremia after ZIKV challenge. It was safe and elicited robust neutralizing antibody titers in healthy non-pregnant women [97] and also induced cross-protective B-cell responses in human against Zika and dengue viruses [98].

Based on the expected safety profile, inactivated virus vaccines including ZPIV adjuvanted with aluminum hydroxide are a potentially favored platform for vaccinating pregnant women. Another inactivated ZPIV vaccine candidate (Takeda’s TAK-426) was found to be well tolerated with an acceptable safety profile and was immunogenic in both flavivirus-naive and flavivirus-primed adults. Based on the safety and immunogenicity profiles of all TAK-426 doses assessed, the TAK-426 was selected for further clinical development [99]. A Vero cell-adapted ZIKV strain (GMZ-002) and a purified inactivated virus (PIV) vaccine were shown to present significantly increased productivity in Vero cells, and IFNAR1-blocked C57BL/6 mice administered with two doses of the PIV were fully protected against lethal challenge. This candidate elicited a robust and persistent protective immunity, therefore representing a promising vaccine candidate for ZIKV [100].

## 7. ZIKV Live Attenuated Vaccine

Live attenuated vaccines generally offer fast and durable immunity, but sometimes with the trade-off of reduced safety [101]. Different candidates have been generated in order to increase safety. A live attenuated vaccine candidate that contains a 10-nucleotide deletion in the 3′ untranslated region of the ZIKV genome (3′UTR 10-del ZIKV) has been shown to be highly attenuated, immunogenic, and protective in the A129 mouse model. Importantly, a single dose of this attenuated vaccine induced sterilizing immunity and a high level of nAbs, completely preventing viremia after challenge and inducing a robust T-cell response in the immunized mice. The attenuated 10-deletion ZIKV was incompetent in infecting mosquitoes after oral feeding of spiked blood meals, representing an additional safety feature for use in non-endemic regions. Collectively, a good balance between immunogenicity and safety warrant further development of this promising live attenuated ZIKV vaccine candidate [102]. 

Another two approaches have been taken to generate live attenuated ZIKV vaccines: (i) Engineering attenuating mutations in an authentic ZIKV isolate, or (ii) generating a chimeric flavivirus using DENV, Japanese Encephalitis virus (JEV), or Yellow Fever virus (YFV) backbones to express ZIKV prM-E genes [103]. For approach (i), a report showed that immunization with the attenuated ZIKV rGZ02a candidate elicits robust inhibitory antibody responses with moderate long-term durability (two medium or high doses of rGZ02a induced robust and persistent (at least 8.5 weeks) responses). rGZ02a, as a booster vaccine, combined with this, greatly improves the protective immunity primed by Ad2-prME, an adenovirus-vectored vaccine expressing ZIKV prM and E proteins, making rGZ02a a promising candidate for a live attenuated ZIKV vaccine [104]. For approach (ii), other studies indicated that chimeric DENV-2 and JEV SA14-14-2 harboring ZIKV prM-E genes protected mice from ZIKV infection after a single-dose vaccination [105,106]. The JEV SA14-14-2 chimeric ZIKV vaccine was also shown to protect non-human primates from ZIKV infection and maternal-to-fetal transmission in mice [105]. Immunization of mice and rhesus macaques with a single dose of a recombinant chimeric ZIKV vaccine candidate expressing the prM-E proteins and based on the licensed Japanese encephalitis live attenuated vaccine SA14-14-2 as the genetic backbone (termed ChinZIKV) elicits robust and long-lasting immune responses and confers complete protection against ZIKV challenge [105]. Following a similar approach, a chimeric ZIKV vaccine deriving from a live attenuated DENV-4 vaccine backbone (rDEN4Δ30, which is safe and induced an antibody response that was broadly neutralizing against genotypically diverse DEN-4 viruses) called rZIKV/D4Δ30-713 has been developed, and is set to undergo phase I clinical trials. It is registered with ClinicalTrials.gov, number NCT03611946, although the preclinical data for the vaccine has not been published yet [107]. These studies provide an alternative vaccine platform in response to the ZIKV emergency, and the safety, immunogenicity, and protection profiles of live attenuated vaccines warrant further clinical development.

## 8. ZIKV Peptide-Based Vaccines

Currently, development of peptide therapeutics against ZIKV (peptide-based vaccines) has attracted rising attention on account of their high safety and low development cost, in comparison to small therapeutic molecules and antibody-based anti-viral drugs [108]. Peptide-based vaccines are usually composed of 20-30 amino acids (aa) made of synthetic B- or T-cells epitopes (class I or class II) that can also be combined, related to infectious and/or chronic diseases including cancers [109]. The use of peptide-based vaccines is a novel approach that implies the identification of different epitopes, both on human cells and virus antigens, in order to induce a specific host immune response to counteract the primary host-pathogen interaction [110]. Advances in immunoinformatic, genetics, bioinformatics, and related sciences have opened the doors to new paradigms in vaccine design [111]. Using characterization techniques and web-based bioinformatics servers, four peptide stretches have been identified in the E protein, being well conserved, surface exposed and predicted to have reasonable epitope binding efficiency [112]. Conserved B- and T-cell epitopes have also been identified for different antigenic proteins [113,114], In-silico 3D modeling and structural validation of the vaccine construct (Conserved B- and T-cell epitopes were merged and further supplemented with β-defensin as an adjuvant, to yield an immunogenic vaccine construct) has been conducted, followed by molecular docking and molecular dynamics simulation studies with human TLR2, which were predicted to stimulate an effective immune response and hence provide protection against both DENV and ZIKV [115]. Other predicted specific epitopes could also play a constructive role in designing a vaccine against ZIKV, including peptides QTLTPVGRL (MHC-I epitope) and IRCIGVSNRDFV (MHC-II epitope), predicted to be highly antigenic among T-cell epitopes [116]. These peptides predicted as epitopes for CD4^+^ and CD8^+^ T cells had the highest MHC-I immunogenicity score and were further tested for interaction against human leukocyte antigen (HLA) molecules [117].In addition, B-cell and IFN-γ epitopes have also been predicted in ZIKV aa sequences, suggesting an important role for the humoral and cell-mediated immune responses acquired by the construct [118]. Therefore, in silico design of multi epitope-based peptide (MEBP) vaccine platforms enables effective prevention against the ZIKV infection. These peptides can be expected to form the basis for a nascent peptide vaccine which, enhanced by incorporation of suitable adjuvants, can elicit immune response against Zika virus infections [112]. These immunoinformatic approaches represent a great tool to initiate the generation of new vaccine candidates for further in vivo validation.

## 9. ZIKV Recombinant Protein Vaccines

As the major target of nAbs, ZIKV E protein has been classically used for vaccine development [119]. To develop subunit vaccines for ZIKV, some research focused on the generation of recombinant ZIKV E protein domain III (EDIII), and the entire ectodomain (E80, which comprises EDI, EDII and EDIII) using both prokaryotic and eukaryotic expression systems, as vaccine candidates. Both constructions were shown to be immunogenic and had protective efficacy in immune competent mice [120]. These products could be further evaluated, either as stand-alone vaccine candidates, or used in a heterologous prime-boost regimen with other forms of ZIKV vaccines. Another strategy has demonstrated that recombinant E proteins generated by using baculovirus or insect cell-produced can induce good immunogenicity in immunized mice [121,122]. These were able to inhibit ZIKV infection by triggering antigen-specific antibody and T-cell response. The use of nanoadjuvants that combine immunostimulatory properties and delivery systems can improve the efficacy of recombinant protein-based vaccines [123]. The study shows that immunization with zEDIII together with saponin-based nanoadjuvant IQB-80 from *Q. brasiliensis* is capable of significantly improving immune responses against ZIKV in mice, which identified saponin-based delivery systems as an adequate adjuvant for recombinant ZIKV vaccines and has important implications for recombinant protein-based vaccine formulations against other flaviviruses [124]. While antibodies targeting the envelope glycoprotein can neutralize the virus, they carry the risk of ADE of disease when they are subneutralizing [125]. In contrast, antibodies generated against the NS1 protein can be protective without eliciting ADE [81], as NS1 is the most conserved antigen among different serotypes. In early studies, purified NS1 protein was studied as a subunit vaccine candidate for many flaviviruses, such as YFV, DENV, and JEV [126,127,128]. Therefore, NS1 could also be a promising vaccine candidate against ZIKV.

## 10. Anti-ZIKV Monoclonal Antibodies Vaccines

Given the limitations on vaccine design against ZIKV infection, passive immunization by using monoclonal antibodies (MAbs) is consequently one of the most thoroughly studied strategies to treat the infection [129,130]. Additional therapeutics under investigation include anti-ZIKV MAbs, that have been shown to neutralize infection in vitro, as well as protect against morbidity in mouse models of ZIKV infection [43]. The anti-E80 and anti-EDIII sera were found to potently neutralize ZIKV infection in vitro [122,131], and passive transfer of either anti-E80 or anti-EDIII sera protected recipient mice against lethal ZIKV challenge [122]. Other approaches, such as high-throughput antibody isolation, have contributed to a better understanding of the B-cell responses elicited following infection and/or vaccination [132]. The isolation of potent nAbs, coupled with detailed examination of their properties at the molecular level, have provided pivotal insights related to immunogen design or, ultimately, cross-flavivirus ZIKV vaccines [133]. The vast majority of these antibodies neutralize flaviviruses by locking E dimers in the pre-fusion conformation and preventing structural rearrangements necessary for E fusogenic activity [134]. Engineering of these nAbs can further enhance their potency and breadth as well as improve their pharmacokinetic properties [135], so that a single infusion could confer protection over several weeks. However, most of the MAbs tested to date have shown a weak response against ZIKV which, again, could create the risk of developing ADE related to ZIKV infection [136,137]. However, monoclonal antibodies targeting the Zika virus nonstructural NS1 protein have been shown to be protective without inducing ADE of disease [138]. The protective efficacy of NS1-targeted MAbs may be linked to their epitope recognition [139]. In addition, MAbs against the NS1 have the potential to suppress ZIKV pathogenicity, illustrating that NS1-targeted MAbs have multifaceted protective effects and provide insights for the development of NS1-based vaccines and therapeutics [138]. These findings suggest that passive vaccination with antibodies could be a useful strategy to protect against ZIKV infection.

## 11. Anti-ZIKV Mosquito Salivary Protein Vaccines

The majority of the viruses in the genus flavivirus are arboviruses, and an imaginative approach to prevent arboviral diseases is based in the observation that arthropod saliva facilitates transmission of arthropod-borne pathogens, such as DENV, ZIKV, CHIKV and other pathogens [140,141,142,143,144,145]. Arboviruses share a common vector source, transmitted between mammalian hosts via invertebrate vectors (mosquitoes and ticks) [146], whose salivary components are reported to aid viral pathogenesis. Viruses carried within mosquito saliva could more easily initiate host infection by taking advantage of the host’s innate and adaptive immune responses to saliva. Salivary components drive the transition from Th1 (effective, desirable) to Th2 responses by altering cytokine, chemokine, and interferon produced by cells, and specific saliva compounds can also induce autophagy, inhibit T and B lymphocyte proliferation and induce apoptosis [147]. This provides a rationale for creating vaccines against mosquito salivary proteins, rather than against only the virus proteins contained within the saliva. Using salivary proteins of clinically important vector mosquitoes, such as *Aedes aegypti*, might contribute to achieving protection against multiple mosquito-borne viral infections [140].

Saliva components are capable of changing the local immune environment, leading to an increase in flavivirus-susceptible cells at the bite site [148]. A previously undescribed salivary gland (SG) protein (termed neutrophil stimulating factor 1 (NeSt1)) was shown to stimulate neutrophils at the mosquito bite site, altering the immune microenvironment and allowing a higher level of early viral replication that triggers ZIKV pathogenesis. Based on these observations, it is possible that a vaccine against NeSt1 might protect people against severe Zika virus infection, based on immunization experiments performed in mice [149]. Another mosquito salivary protein, *A. aegypti* bacteria-responsive protein 1(AgBR1), could induce inflammatory responses at the bite site. It has been described that passive immunization with AgBR1 antiserum and active immunization with recombinant AgBR1 protein adjuvanted with aluminum hydroxide partially protected mice from a mosquito-borne ZIKV infection [150,151], suggesting that AgBR1 may be used as another target for vaccine development against ZIKV. Further research showed that passive immunization with a combination of AgBR1- and NeSt1-sera enhanced survival and reduced the viral burden in blood, thereby protecting mice from mosquito-borne ZIKV infection [152]. There are also many other mosquito salivary proteins that could promote ZIKV transmission, such as LTRIN by its interaction with the lymphotoxin β receptor or AaVA-1 activating autophagy pathways [153,154]. Taken together, these findings suggest that targeting a combination of mosquito saliva proteins could be an interesting approach for vaccine development to help prevent mosquito-borne ZIKV infection.

## 12. Conclusions

Here, we summarize some information on vaccine candidates on different vaccine platforms in clinical trials (Table 1). Among those, several vaccine candidates against ZIKV have completed phase I or phase II trials and have been shown to be safe, well tolerated, and immunogenic in healthy adult volunteers. In addition, many Zika vaccine candidates have been evaluated from different platforms (Table 2), and these viable Zika vaccine candidates should continue their development. ZIKV has become endemic and new cases of Zika congenital syndrome continue to be reported in endemic areas. It is very likely that we will face another large Zika epidemic in the next few years as herd immunity decays. Therefore, it is critical for Zika vaccine developers to be ready to activate phase III trials and ramp up vaccine production, in case another epidemic emerges. Vaccine developers should also consider coordinating strategies to use dengue and Zika vaccines to maximize the immunogenicity of both vaccines and to reduce the risks associated with deleterious DENV and ZIKV cross-reactive antibody interactions.

In summary, there is a lot that is still unknown about ZIKV, and the occurrence of more prominent epidemics caused by other pathogenic microorganisms has slowed down the advance of ZIKV-related research. The cellular and animal models available have shed light on how the virus acts, why it acquired a more aggressive nature in the recent epidemics and how to potentially combat it more effectively, but there is still a lot of progress to be made in the development of effective vaccines, and both organoid cultures and nanotechnology compounds can pave the way to achieve them.

## Figures and Tables

**Table 1:** Vaccine candidates on different vaccine platforms in clinical trials.

Vaccine Platforms	Condition or Disease	ClinicalTrials.Gov Identifier	Intervention/Treatment	Phase	Recruitment Status	Sponsors and Collaborators
DNA Vaccine	Zika Infection	NCT02840487 [41]	Biological: VRC-ZKADNA085-00-VP	Phase 1	Completed	National Institute of Allergy and Infectious Diseases (NIAID)
	Zika Infection	NCT02996461 [41]	Biological: VRC-ZKADNA090-00-VP	Phase 1	Completed	NIAID
	Zika virus	NCT02809443 [37]	Biological: GLS-5700	Phase 1	Completed	GeneOne Life Science, Inc. (Seoul, Korea), Inovio Pharmaceuticals
	Zika Virus	NCT03110770 [41]	Biological: VRC-ZKADNA090-00-VP VRC-PBSPLA043-00-VP	Phase 2	Completed	NIAID
mRNA Vaccine	Zika Virus	NCT04064905 [58]	Biological: mRNA-1893 Placebo	Phase 1	Completed	ModernaTX, Inc. (Cambridge, UK)
	Zika Virus	NCT03014089	Biological: mRNA-1325 Placebo	Phase 1	Completed	ModernaTX, Inc.
	Zika Virus	NCT04917861 [59]	Biological: mRNA-1893 Placebo	Phase 2	Active, Not recruiting	ModernaTX, Inc.
Viral Vector Vaccine	Zika Virus	NCT04015648	Biological: ChAdOx1 Zika	Phase 1	Completed	University of Oxford
Purified Inactivated Vaccine	Zika Virus	NCT02937233	Biological: Zika Virus Purified Inactivated Vaccine Placebo	Phase 1	Completed	Kathryn Stephenson, Walter Reed Army Institute of Research (WRAIR), NIAID
	Zika Virus	NCT02963909	Biological: IXIARO Placebo YF Vax 17D Strain Zika Virus Purified Inactivated Vaccine (ZPIV)	Phase 1	Completed	NIAID
	Zika Virus	NCT03008122	Biological: Zika Virus Purified Inactivated Vaccine (ZPIV) Placebo	Phase 1	Completed	NIAID
Live Attenuated Vaccine	Zika Virus	NCT03611946 [107]	Biological: rZIKV/D4Δ30-713 Placebo	Phase 1	Completed	NIAID

**Table 2:** Zika vaccine candidates on different vaccine platforms.

Vaccine Platforms	Principle to Generate	Vaccine Candidates	Characteristics of Vaccine Platform
DNA Vaccine	Cloning an antigen(s) from a pathogen into a DNA plasmid	VRC5288 [41], VRC5283 [41], GLS-5700 [43]	Quickly designed and manufactured
mRNA Vaccine	mRNA can be synthesized with virtually any desired sequence	ZIKV prM-E mRNA-LNP [53], mRNA (SAM) [54], mRNA-1893 [58]	Simplified, flexible, and fast vaccine production platform
Viral Vector Vaccine	Ad vectors: insert a ZIKV prM-E gene expression cassette into human Ad vectors	Ad5-prM-E [65], Ad26.ZIKV.001 [69], ChAdOx1-EDIII [71]	Ad vectors are amenable to rapid, inexpensive scale-up since they are easy to engineer
	Poxvirus-based vectors: MVA is an attenuated poxvirus that is frequently used as viral vector	MVA-BN-Filo [74], MVA-ZIKV-NS1 [79]	Poxvirus-based vectors have a large payload capacity, potential for cold chain-independent distribution.
	rVSV: expressing ZIKV prM-E-NS1 for protection against ZIKV infection and highlights an important role for NS1 in ZIKV-specific cellular immune responses	ZIKV prM-E-NS1 [80]	Antibody response against the Zika virus NS1 protein is long-lasting and functionally active
	SCV: is derived from the Copenhagen strain of VACV	SCV-ZIKA/CHIK [72]	As a multi-disease vaccine platform capable of delivering multiple large immunogens
VLPs vaccines	Based on the use of structural proteins, can be achieved by different approaches including DNA (e.g., NIAID/VRC), mRNA (e.g., Moderna)	VLPs produced in HEK293 using the prM and E structural proteins [87], VLPs co-expression of the prM-E, prM-E-NS1, C-prM-E, and NS2B/NS3 in human cells [88], Zika-VLP [92]	A cost-effective, highly protective and safe ZIKV vaccination strategy, some of them representing a safer vaccine for preventing ADE
Purified Inactivated Vaccine	Consisting of virus particles, bacteria, or other pathogens that have been grown in culture	ZPIV [96], Takeda’s TAK-426 [99], GMZ-002-PIV [100]	Have a long track record of safety in both pregnant women and fetuses
Live Attenuated Vaccine	Reducing the virulence of a pathogen, but still keeping it viable	3′UTR 10-del ZIKV [102], ZIKV rGZ02a [104], ChinZIKV [105], rZIKV/D4Δ30-713 [107]	Offer fast and durable immunity, but sometimes with the trade-off of reduced safety
Peptide-based Vaccines	Composed of 20–30 aa made of synthetic B- or T-cells epitopes that can also be combined	Predicted specific epitopes including: human TLR2 [115], MHC-I, MHC-II [116] and MEBP [112]	High safety and low development cost
Recombinant Protein Vaccines	Using both prokaryotic and eukaryotic expression systems to generate recombinant ZIKV EDIII, and the E80, which comprises EDI, EDII and EDIII	zEDIII with saponin-based nanoadjuvant IQB-80 from Q. brasiliensis [124], Zika-NS1	Immunogenic and show protective efficacy
Monoclonal Antibodies Vaccine	A form of immunotherapy that uses monoclonal antibodies (mAbs)	Anti-E80 and anti-EDIII sera [122], NS1-targeted MAbs [139]	One of the most thoroughly studied strategies to treat the infection and NS1-targeted MAbs have multifaceted protective effects
Anti-ZIKV Mosquito Salivary Protein Vaccines	Creating vaccines against mosquito salivary proteins	NeSt1 [149], AgBR1 [150], LTRIN [153]	Arthropod saliva facilitates transmission of arthropod-borne pathogens, targeting a combination of mosquito saliva proteins could be an interesting approach for vaccine development
